# Functional evaluation of *PDGFB*-variants in idiopathic basal ganglia calcification, using patient-derived iPS cells

**DOI:** 10.1038/s41598-019-42115-y

**Published:** 2019-04-05

**Authors:** Shin-ichiro Sekine, Masayuki Kaneko, Masaki Tanaka, Yuhei Ninomiya, Hisaka Kurita, Masatoshi Inden, Megumi Yamada, Yuichi Hayashi, Takashi Inuzuka, Jun Mitsui, Hiroyuki Ishiura, Atsushi Iwata, Hiroto Fujigasaki, Hisamitsu Tamaki, Ryusei Tamaki, Shinsuke Kito, Yoshiharu Taguchi, Kortaro Tanaka, Naoki Atsuta, Gen Sobue, Takayuki Kondo, Haruhisa Inoue, Shoji Tsuji, Isao Hozumi

**Affiliations:** 1 Laboratory of Medical Therapeutics and Molecular Therapeutics, Gifu Pharmaceutical University, Gifu, Japan; 2 Center for iPS Cell Research and Application, Kyoto University, Kyoto, Japan; 3 Department of Biochemistry, Institute of Biomedical and Health Sciences, Hiroshima University, Hiroshima, Hiroshima Japan; 4 Department of Neurology, The University of Tokyo, Tokyo, Japan; 5 Department of Neurology and Geriatrics, Gifu University Graduate School of Medicine, Gifu, Japan; 6 Department of Neurology, Tokyo Metropolitan Bokuto Hospital, Tokyo, Japan; 7 Department of Pediatrics, Tokyo Metropolitan Bokuto Hospital, Tokyo, Japan; 8 Department of Neuropsychiatry, Kyorin University Hospital, Tokyo, Japan; 9 Department of Neurology, Toyama University Hospital, Toyama, Japan; 10 Department of Neurology, Nagoya University, Nagoya, Japan; 11 iPSC-based Drug Discovery and Development Team, RIKEN BioResource Research Center, Kyoto, Japan; 12 Medical-risk Avoidance based on iPS Cells Team, RIKEN Center for Advanced Intelligence Project, Kyoto, Japan

## Abstract

Causative genes in patients with idiopathic basal ganglia calcification (IBGC) (also called primary familial brain calcification (PFBC)) have been reported in the past several years. In this study, we surveyed the clinical and neuroimaging data of 70 sporadic patients and 16 families (86 unrelated probands in total) in Japan, and studied variants of *PDGFB* gene in the patients. Variant analyses of *PDGFB* showed four novel pathogenic variants, namely, two splice site variants (c.160 + 2T > A and c.457−1G > T), one deletion variant (c.33_34delCT), and one insertion variant (c.342_343insG). Moreover, we developed iPS cells (iPSCs) from three patients with *PDGFB* variants (c.160 + 2T > A, c.457−1G > T, and c.33_34 delCT) and induced endothelial cells. Enzyme-linked immunoassay analysis showed that the levels of PDGF-BB, a homodimer of PDGF-B, in the blood sera of patients with PDGFB variants were significantly decreased to 34.0% of that of the control levels. Those in the culture media of the endothelial cells derived from iPSCs of patients also significantly decreased to 58.6% of the control levels. As the endothelial cells developed from iPSCs of the patients showed a phenotype of the disease, further studies using IBGC-specific iPSCs will give us more information on the pathophysiology and the therapy of IBGC in the future.

## Introduction

Idiopathic basal ganglia calcification (IBGC), also known as Fahr’s disease^1^ or recently referred to as primary familial brain calcification (PFBC)^2^, is a rare and intractable disease. It is characterized by abnormal deposits of minerals including calcium (Ca) in the basal ganglia and other brain regions, such as the thalamus and cerebellum. Most cases are idiopathic in Japan. Regarding the diagnosis of IBGC, other secondary causes of calcification should be excluded^1,2^. The diverse clinical manifestations of IBGC include parkinsonism, cerebellar symptoms, cognitive impairment, psychosis, seizures, and chronic headache^2^. The following causative genes for familial IBGC (FIBGC) have been successively identified: *SLC20A2* (IBGC1 [previously called IBGC3 and now referred to as IBGC1])^3^, *PDGFRB* (IBGC4)^4^, *PDGFB* (IBGC5)^5^, *XPR1* (IBGC6)^6^ as autosomal dominant traits, and *MYORG*^7^ as an autosomal recessive trait. *Myorg* mRNA is expressed in astrocytes, and this may provide new insights on the mechanism underlying brain calcification^6^.

Several studies, including our studies, have shown that *SLC20A2* variants are the most frequent in patients with IBGC in many countries^2,8–10^. *PDGFRB* variants encoding platelet-derived growth factor (PDGF) receptor-β (PDGFRβ), *PDGFB* encoding its ligand, and *XPR-1* encoding a transporter which exports inorganic phosphorus (Pi) out of the cells, have been reported in the past several years. In this study, we conducted a nationwide survey for *PDGFB* variant in Japanese patients with IBGC.

PDGF is a dimeric glycoprotein which is composed of two subunits from the four components: A, B, C, and D. PDGFR, the receptor of PDGF, is classified as a receptor tyrosine kinase. PDGF-B is expressed in vascular endothelial cells and neurons in the brain^5,11^. PDGF-BB, a homodimer of PDGF-B, stimulates pericytes which are abundant in the brain^12^.

The specific treatment has not been found yet for patients with IBGC, including those with *PDGFB* variants. Mice models carrying hypomorphic human *pdgfb* alleles have been developed and showed calcium deposits in the brain^5^. However, the genetic pathophysiological mechanisms and the calcification sites in mice were different from those of humans^5^. We produced iPS cells (iPSCs) from a patient with *SLC20A2* variant^13^. New models of IBGC, including iPSCs, should be developed for further investigation, especially for drug treatment. Then, we developed iPSCs from patients with *PDGFB* variants and induced the endothelial cells. They mainly produce PDGF-BB which stimulates the pericytes in the brain. The breakdown of the pathway due to the loss of function is thought to cause the disruption of pericytes^14^ and blood brain barrier (BBB)^15–18^. The decrease in the production of PDGF-BB in endothelial cells may be a target for the therapy for patients with *PDGFB* variants.

We have observed higher levels of Pi in CSF not only in patients with *SLC20A2* variants but in other patients without variants than those of controls^19^. The role and presence of Pi is critical in the pathogenesis of IBGC in addition to Ca. This shows the disturbance in the intracellular uptake of Pi in patients with IBGC. PDGF-BB has been reported to stimulate the activation of a Pi transporter, PiT-1, which is encoded by *SLC20A1*, and increases the intracellular uptake of Pi in the vascular smooth muscle cells^20^. In this study we evaluated the level of PDGF-BB in sera and culture media of endothelial cells derived from iPSCs of humans.

## Results

### Variant analysis

**Figure 1:**
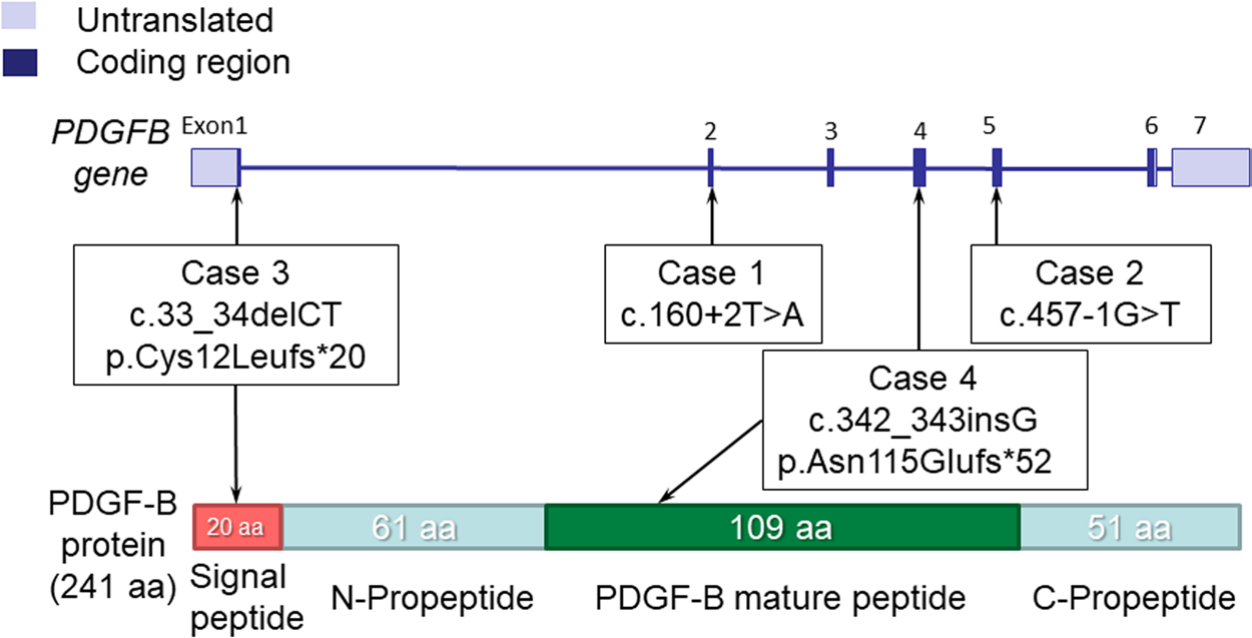
*PDGFB* variants and PDGF-B protein. (Upper) Schematic diagrams of *PDGFB* with variants. Four *PDGF* variants (one insertion variant in exon 4 [c.342_343insG], one deletion variant in exon 1 [c.33_34delCT], and two splice site variants in exon 2 [c.160 + 2T > A] and exon 5 [c.457−1G > T]) were found in the *PDGFB* gene. (Lower) Schematic structure of the PDGF-B protein with variants. aa = amino acid.

We sequenced the PDGFB gene in 70 sporadic patients with IBGC and 16 families (86 unrelated probands in total) in Japan, using the Sanger method sequencing of all coding regions. Four variants s were found in *PDGFB*: two variants at the splice junctions (Case 1: c.160 + 2T > A; Case 2: c.457−1G > T), one deletion variant (Case 3: c.33_34delCT), and one insertion variant (Case 4: c.342_343insG) (Table 1, Fig. 1). Electropherograms indicated that all variants were heterozygous (Supplementary Fig. 1). None of the variants were found in an in-house exome sequencing data set (358 Japanese control participants), dbSNP build 151 (https://www.ncbi.nlm.nih.gov/projects/SNP), Genome Aggregation Database (gnomAD) version 2.0 (http://gnomad.broadinstitute.org), Japanese Multi Omics Reference Panel (jMorp) (https://jmorp.megabank.tohoku.ac.jp), and the Human Gene Mutation Database (HGMD) professional 2018.2 (http://www.hgmd.cf.ac.uk). When confined to FIBGC, two of the 16 (12.5%) families showed *PDGFB* variants. In contrast, two of the 70 (2.9%) patients with sporadic IBGC carried *PDGFB* variants.

**Table 1:** Clinical features of the four individuals (probands) with *PDGFB* variants.

	Case 1	Case 2	Case 3	Case 4
Variant	c.160 + 2T > A	c.457−1G > T	c.33_34delCT	c.342_343insG
			p.Cys12Leufs*20	p.Asn115Glnfs*52
Zygosity	Hetero	Hetero	Hetero	Hetero
Exon	2	5	1	4
**Proband’s information**				
Age at the detection of calcification, y	57	14	71	53
Age at onset, y	57	10	70	56
Symptoms	anxiety depression	headache school refusal	dizziness	dementia avolition
**Neurological findings**				
cognitive impairment (MMSE)	28	NE	23	13
pyramidal sign	−	−	−	−
extrapyramidal sign	−	−	+	−
cerebellar sign	+	−	−	−
**Family’s information (except for the proband)**				
Number of other individuals with calcification	1	2	NE	NE
Number of other individuals with confirmed variants	1	2	−	−
Number of other symptomatic individuals	1	1	−	−
Other symptoms (number) in the family	panic disorder (1)	MR (1)	−	−

Abbreviations: NE = not examined, N/A = not applicable, MMSE = mini-mental state examination, MR = mental retardation.

### Clinical manifestations and variants in the probands and their families Familial cases

#### Case 1 (in family 1)

**Figure 2:**
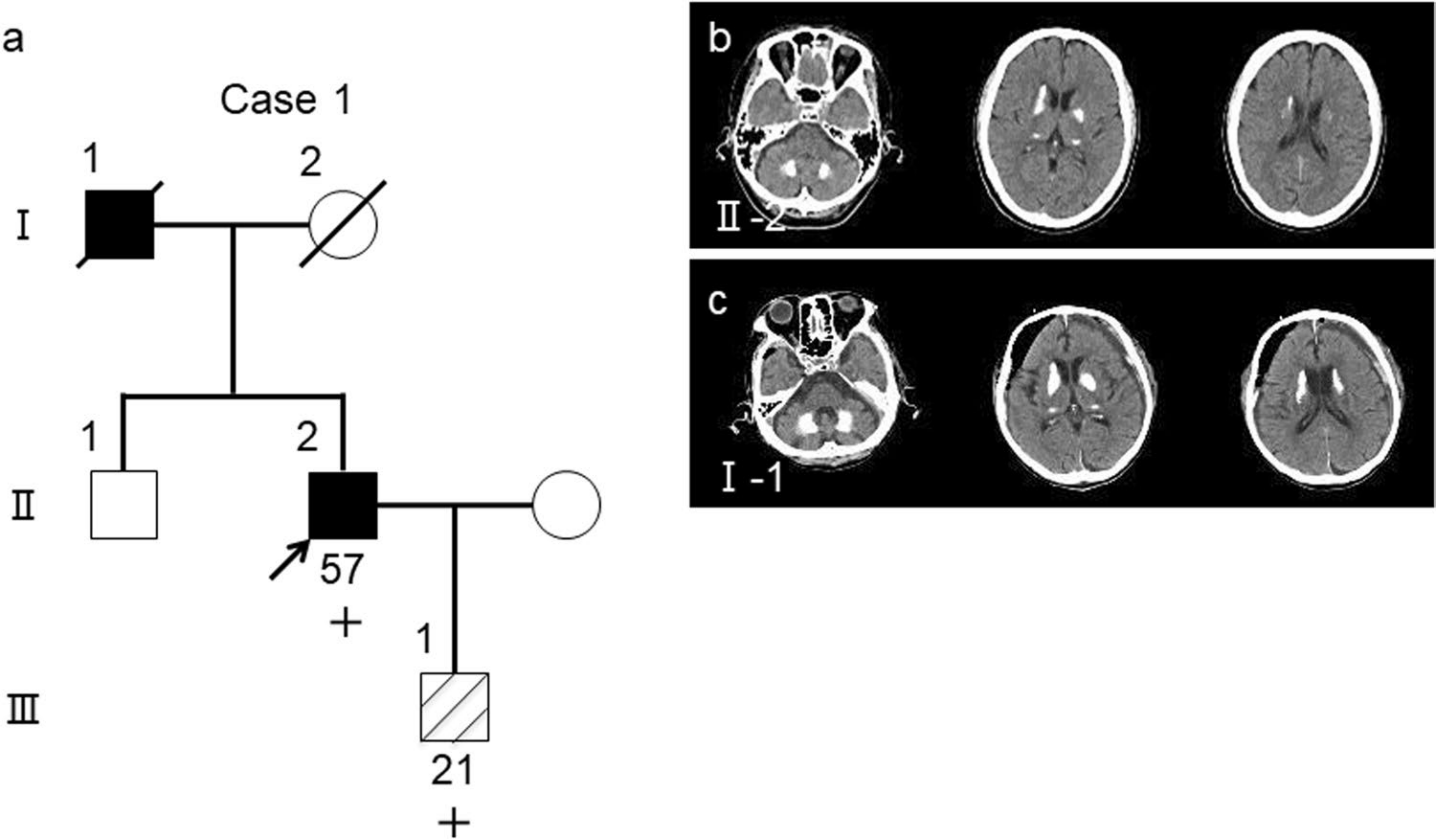
FIBGC (Case 1 *in family 1*). (**a**) Pedigree of *family 1*. The arrow indicates the index subject. Filled symbols represent patients with brain calcification. Participants’ ages are shown under the symbols in the pedigree of those whose data were available. The symbols + and − indicate variant carriers and noncarriers, respectively, as determined via genetic analysis. The striped symbol represents a variant carrier, although the CT image was not available for the study. (**b**) CT images of the proband (II-2). (**c**) CT images of the proband’s father with variant (I-1). Subdural hematoma was also identified on CT scan.

The proband (Fig. 2a; II-2) in the family was a 57-year-old man who was experiencing depression, anxiety, and mild cognitive impairment for 3 years. His neurologic examination findings revealed mild cerebellar ataxia (Table 1). Computed tomography (CT) images revealed marked calcification in the bilateral globus pallidus, caudate nuclei, pulvinar thalami, and dentate nuclei (Fig. 2b). His deceased father (Fig. 2a; I-1) had dementia. Moreover, CT images of his father showed similar findings to those of the proband (Fig. 2c). The son of the proband (Fig. 2a; III-1) had been treated for panic disorder since his teen years. He had the same variant as that of the proband. However, we have not confirmed brain calcification on CT images of III-1.

#### Case 2 (in family 2)

**Figure 3:**
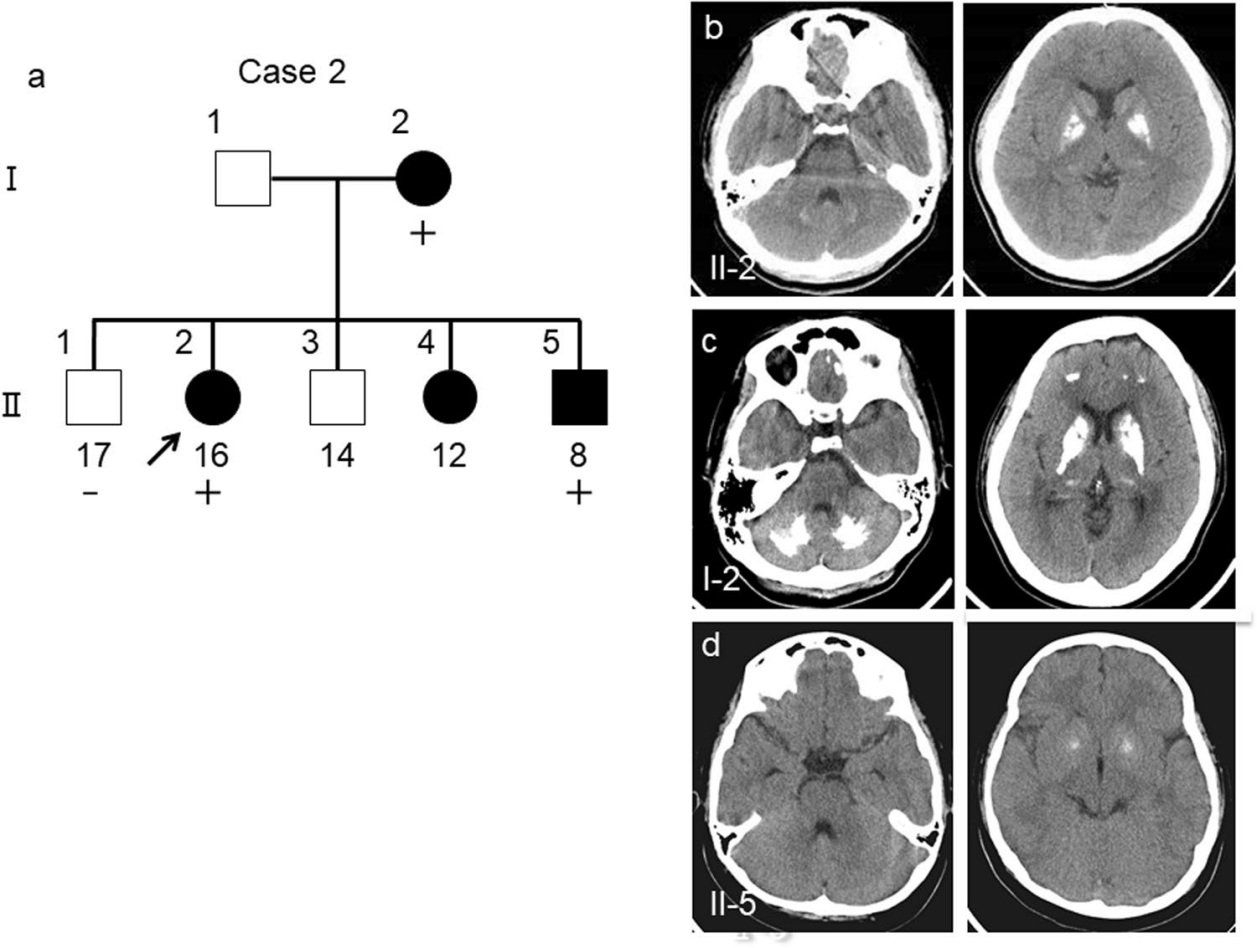
FIBGC (Case 2 *in family 2*). (**a**) Pedigree of *family 2*. The arrow indicates the index subject. Filled symbols represent patients with brain calcification. Participants’ ages are shown under the symbols in the pedigree. The symbols + and − indicate variant carriers and noncarriers, respectively. CT images of the proband (II-2) (**b**). Computed tomography (CT) image of her mother (I-2) with the variation (**c**). CT image of her younger brother (II-5) with the variation. The calcification of II-5 was mild (**d**). The calcification on CT images of the proband’s mother (I-2, C) was prominent compared to that of her children (II-2, B) and (II-5, D).

The proband (Fig. 3a; II-2) was a 16-year-old woman. She presented with headaches and had been refusing to attend school since 10 years old. Her neurological examination findings were normal (Table 1). CT images revealed spotty calcification in the bilateral globus pallidus and caudate nuclei and mild calcification in the thalamus, subcortical white matter, and dentate nuclei (Fig. 3b). Her mother (Fig. 3a; I-2) had the same variant, and she also presented with headaches and prominent calcification in the bilateral globus pallidus, caudate nuclei, thalamus, dentate nuclei, and subcortical white matter (Fig. 3c). On the basis of these results, the calcification was believed to progress with age. Although her third brother (Fig. 3a; II-5) was asymptomatic, he showed mild calcification in the globus pallidus on CT images obtained when he encountered a traffic accident (Fig. 3d). Considering his age, this calcification was pathologic (total calcification score = 6)^21^. The calcification in other regions of the brain, including the dentate nuclei of the cerebellum, could not be detected. DNA analysis revealed the same variant (Fig. 3a; II-5). Her eldest brother did not present with the variant. Thus, no calcification was observed on CT images (data not shown) (Fig. 3a; II-1). Her younger sister was in a nursing institution because of mental retardation, and a detailed clinical information about her sister was not available (Fig. 3a; II-4).

### Sporadic cases

#### Case 3

**Figure 4:**
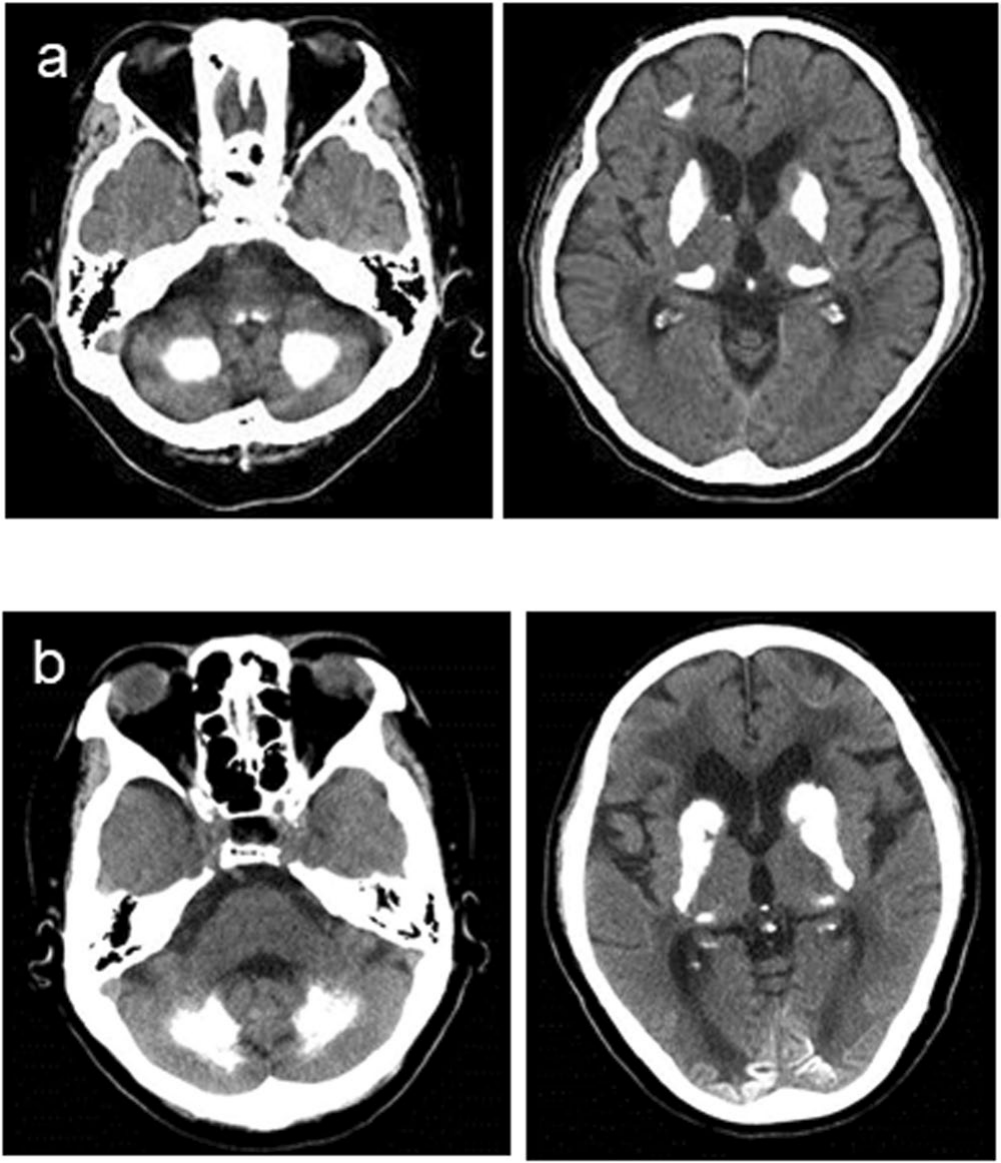
CT images. (**a**) Computed tomography (CT) images of Case 3 with PDGFB deletion variant (c.33_34delCT). (**b**) CT images of Case 4 with PDGFB insertion variant (c.342_343insG).

The patient was a 71-year-old man who often presented with dizziness since 70 years old. He had mild cognitive impairment (mini-mental state examination [MMSE] score: 23) and mild bradykinesia based on his neurological examination (Table 1). Brain CT images showed calcified areas in the bilateral globus pallidus, caudate nuclei, pulvinar thalami, dentate nuclei, and subcortical and periventricular regions (Fig. 4a).

#### Case 4

The patient was a 60-year-old woman who had memory impairment accompanied with avolition since 50 years old. At age of 53, brain calcification was detected on CT images. She developed dementia at age 56 and underwent a medical examination at age 60. Her MMSE score was 13. She presented with impaired postural reflexes and signs of frontal lobe impairment, and she did not have any other neurological problems except for postural instability (Table 1). Brain CT images revealed calcification in the bilateral globus pallidus, caudate nuclei, pulvinar thalami, dentate nuclei, and subcortical white matter as well as atrophy in the medial temporal lobe (Fig. 4b). Furthermore, single photon emission computed tomography and positron emission tomography (PET) images showed decreased signals in the striatum and temporal and parietal lobes, and [^11^C] Pittsburgh compound B (PiB) retention was observed on [^11^C] PiB PET (data not shown), which suggests that she also had Alzheimer’s disease. Unfortunately, the patient had not come to our hospital in the past. Then, we could not measure the level of PDGF-BB in the serum and produce iPS cells from the patient.

### Effects of variants on *PDGFB* mRNA splicing

**Figure 5:**
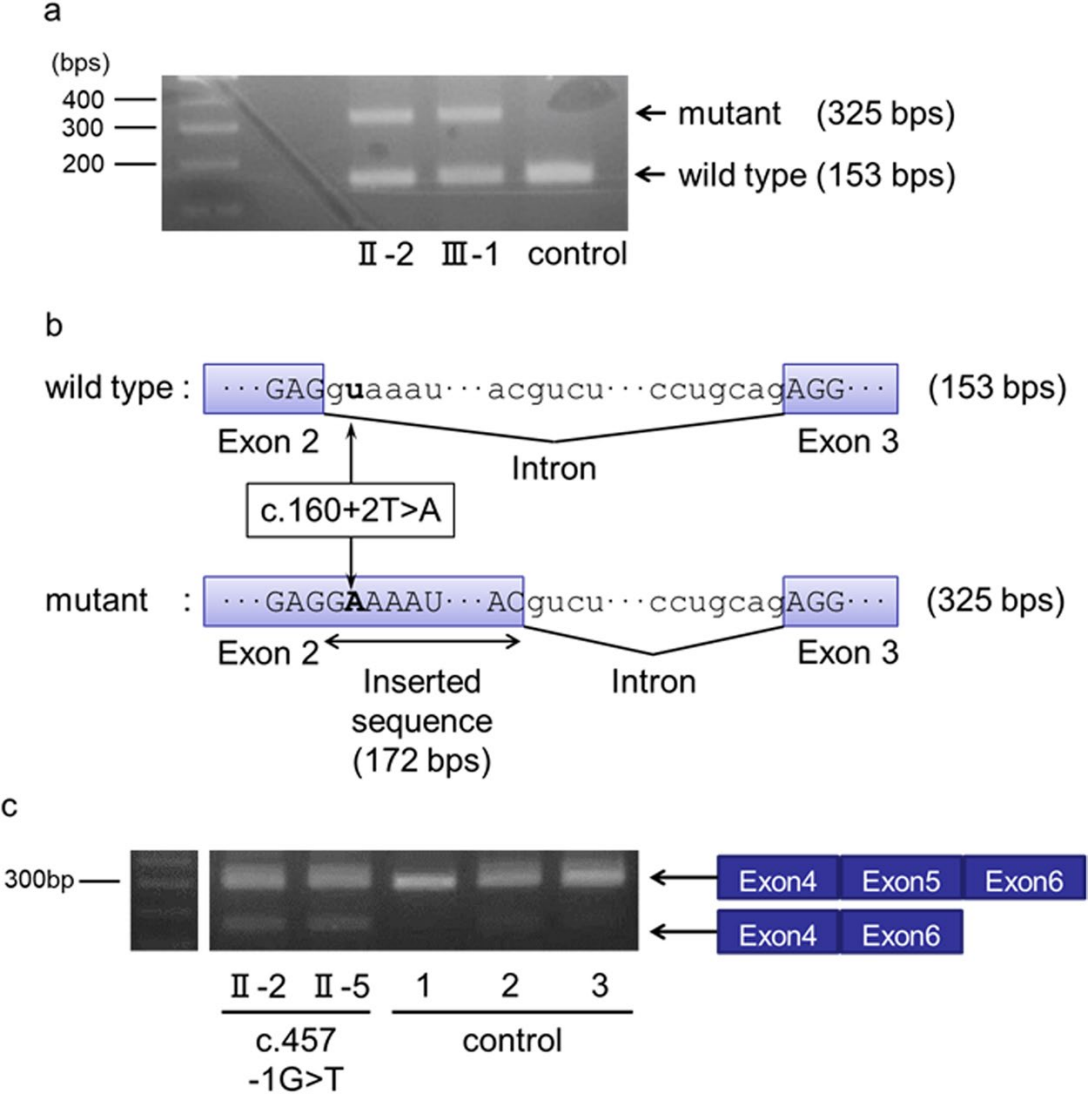
Patients with FIBGC who presented with *PDGFRB* splicing site variant. (**a**) (Case 1) Splicing patterns of PDGFB mRNA with a splicing site variant. mRNA from the patients’ blood was reverse transcribed, and cDNA sequences between exons 2 and 3 (including c.160 + 2T > A) were amplified via polymerase chain reaction (PCR). (**b**) (Case 1) Sequence of PDGFB mRNA with intron sequence that was removed. PCR products were purified and sequenced. Uppercase and lowercase letters indicate exon and intron sequences, respectively. The boxes represent differentially spliced exons. The boldface in red indicates the splice site variant (c.160 + 2T > A). (**c**) (Case 2) Splicing patterns of PDGFB mRNA with splice site variant. The mRNA from the patients’ blood was reverse transcribed, and the cDNA sequences between exons 4 and 6 (including c.457−1G > T) were amplified via PCR. The sequence of PDGFB mRNA with intron sequence that was removed. PCR products were purified and sequenced. The upper bands indicate the wild-type products, and the lower bands indicate the variant products that lack exon 5. The exon 5 was removed from PDGFB mRNA.

To confirm the effect of *PDGFB* variants (Case 1, c.160 + 2T > A; Case 2, c.457−1G > T) on pre-mRNA splicing at the splice sites, the sequences between exons (Case 1, exons 2–3; Case 2, exons 4–6) were amplified via reverse transcription polymerase chain reaction (RT-PCR), followed by direct sequencing. The variant (c.160 + 2T > A) in Case 1 showed that the PCR products corresponded to the predicted lengths containing 172 bps of the unspliced intron between the exons (Fig. 5a,b). For the variant (c.457−1G > T) in Case 2, the proband (Fig. 3a; II-2) and her third brother (Fig. 3; II-5) had lower-shifted PCR products corresponding with the transcript that lacks exon 5. By contrast, the cDNA of the controls did not have the shifted PCR products (Fig. 5c).

### Levels of PDGF-BB protein in the sera of patients with *PDGFB* variants, those with *SLC20A2* variants, and healthy controls

PDGF-BB, a major dimeric glycoprotein, is composed of two subunits of PDGF-B. To determine the effect of the variants on PDGF-BB protein levels, we measured the PDGF-BB levels in the blood of patients with IBGC and healthy controls. Moreover, we measured those of patients with *SLC20A2* variants to clarify the presence of convergence into a common pathogenic mechanism. Enzyme-linked immunoassay (ELISA) analysis showed the PDGF-BB protein levels in the blood sera of three patients except for Case 4 with *PDGFB* variants. Data of Case 4 were not available. The PDGF-BB protein levels of patients with *PDGFB* variants significantly decreased compared with those of the controls (34.0%) and those of patients with *SLC20A2* variant (40.0%) (*p* < 0.01 in Student’s t-test) (Table 2).

**Table 2:** Serum levels of PDGF-BB.

		PDGF-BB (pg/mL)	DNA	Protein	PolyPhen-2
***PDGFB***					
Case 1	57/M	1900	c.160 + 2T > A	—	Not applicable
Case 2	16/F	1340	c.457 − 1G > T	—	Not applicable
Case 3	71/M	1730	c.33_34delCT	p.Cys12Leufs*20	Not applicable
Case 4	53/F	NE	c.342_343insG	p.Asn115Glnfs*52	Not applicable
	Average (means ± SD)	1657 ± 287			
SLC20A2*					
Yamada *et al*. Case 1	64/F	4720	c.1909A > C	p.Ser637Arg	Probably damaging
Yamada *et al*. Case 3	69/F	3370	c.344C > T	p.Thr115Met	Probably damaging
Yamada *et al*. Case 5	24/M	4370	c.1399C > T	p.Arg467Xaa	Not applicable
	Average (means ± SD)	4153 ± 700			
**Controls**					
Control 1	24/M	5030	—	—	—
Control 2	29/M	3890	—	—	—
Control 3	33/F	5690	—	—	—
	Average (means ± SD)	4870 ± 910			

Abbreviations: NE = not examined.

*Yamada M *et al*. Neurology 2014; 82: 705–712.

### Endothelial cell differentiation from the iPSCs of patients with *PDGFB* variants

**Figure 6:**
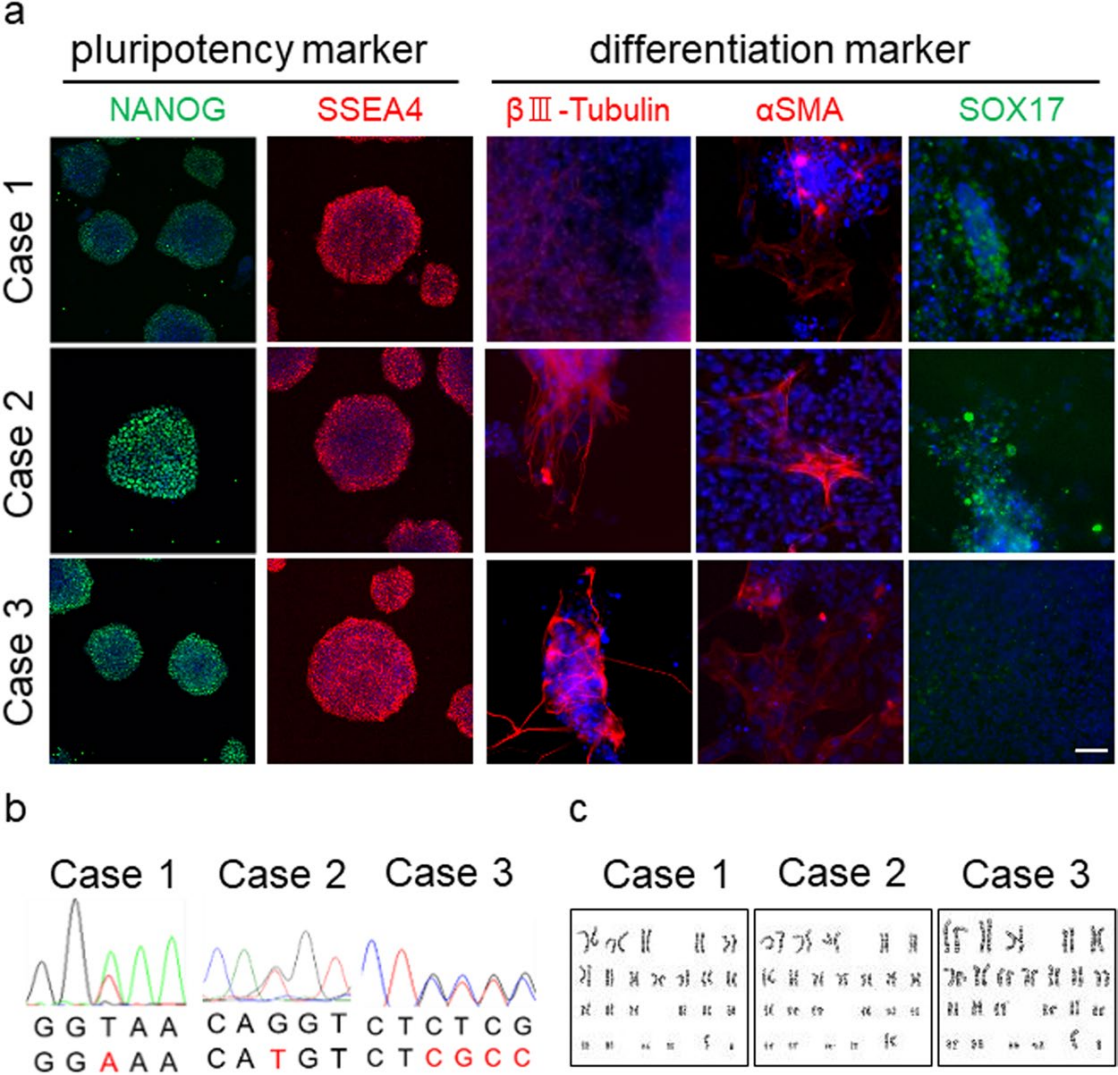
Establishment of iPS cells obtained from patients. (**a**) Immunocytochemical study of iPS cells for the pluripotent marker NANOG, SSEA4, and differentiation markers for three germ layers, namely, βIII-Tubulin, αSMA, and Sox17. (**b**) Sequencing analysis of iPS cells. PDGF-B heterozygous base substitution resulted in variant c.33_34delCT, c.160 + 2T > A, and c.457 − 1G > T. (**c**) G-banded karyotype analysis of iPSCs. The scale bar shows 50 μm.

**Figure 7:**
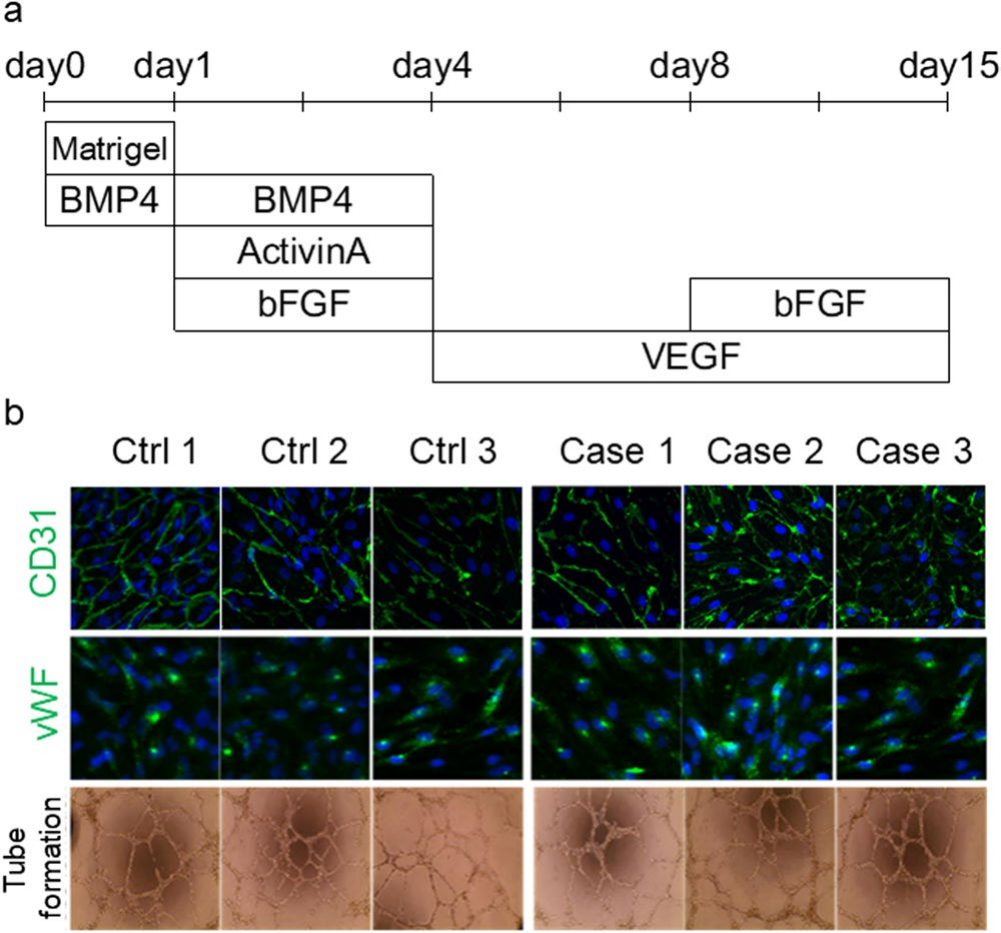
Endothelial cell differentiation from iPSCs obtained from patients. (**a**) Endothelial cell differentiation from iPSCs. (**b**) Immunocytochemical study of endothelial marker CD31 and von Willebrand factor (vWF). Capillary tube formation on Matrigel for iPS-ECs.

**Figure 8:**
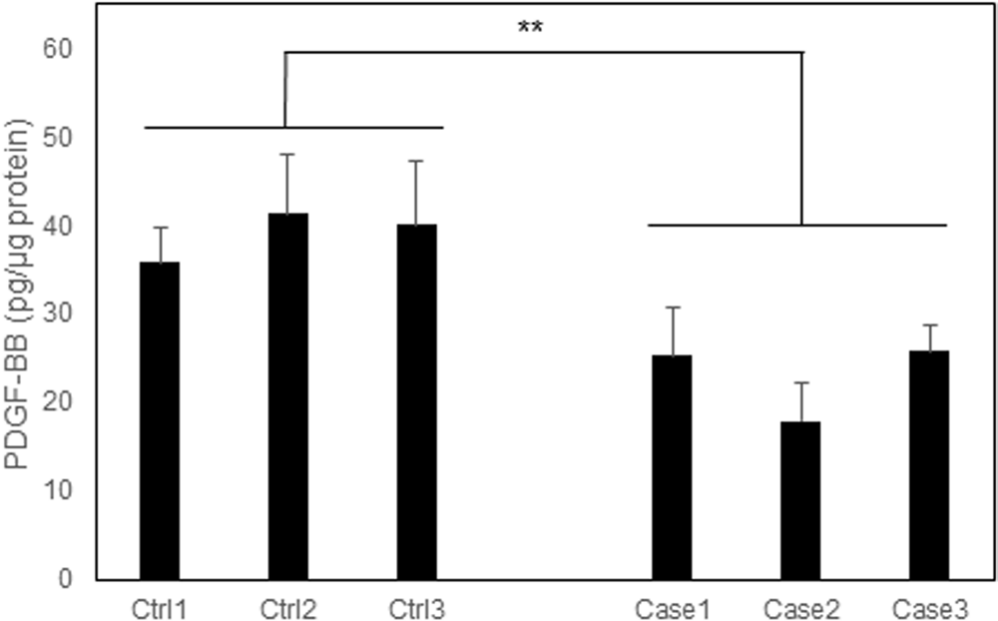
Measurement of PDGF-BB released in the supernatant of iPS-derived endothelial cells using ELISA. iPSC-derived ECs were plated on 96-well plates and cultured for 116 h. Supernatant from iPSC-derived endothelial cells were measured using ELISA. Data were presented as means ± SD. Statistical significance was determined using Student’s t-test (**p < 0.01).

The establishment of iPSCs had been successfully confirmed in *in vitro* differentiation study (Fig. 6a), genotyping analysis (Fig. 6b), and karyotype analysis (Fig. 6c). Differentiation into endothelial cells had been successfully performed from iPSCs according to the method with modified protocol^22^ (Fig. 7a). Their differentiation was identified via immunocytochemical study, and tubular formation was detected via morphological study (Fig. 7b). PDGF-BB protein levels in the culture media of the patients (Cases 1, 2, and 3) (22.9 ± 4.45 pg/μg protein) were significantly lower (58.6%) than those of healthy controls (39.1 ± 2.93 pg/μg protein) (*p* < 0.01 Student’s t-test). (Fig. 8).

## Discussion

We have detected four novel variants of *PDGFB* in patients with IBGC. All variants are interpreted to be pathogenic according to ACMG/AMP variant classification guideline^23,24^ because they resulted in frameshifts or abnormal splicing leading to dynamic changes in protein translation. Several studies have reported pathogenic variants in *PDGFB*^5,9,25–28^. Most variants are thought to act as null variants. Some missense variants are predicted to act through loss of function mechanisms^29^. Species differences and region-specific susceptibility should be also examined in the future. In this study, in patients with *PDGFB* variants, PDGF-BB levels in the blood sera were down to less than half of those of controls and patients with *SLC20A2* variants. The reduced PDGF-BB levels are assumed to be due to *PDGF-B* variant alleles which were expected as null variants.

In the survey, we identified four novel variants in *PDGFB* in 105 Japanese patients with IBGC. Among the 16 families with FIBGC, the variant frequency of *PDGFB* was 12.5% [2 of 16 families]. The variant frequency in this study is similar to the frequencies (19%, [6 of 32 families]) in the first report^5^. Presently, in Japan, the main causative gene for FIBGC is *SLC20A2* (31.3%, [5 of 16 families]) and the second causative gene is *PDGFB* (12.5%). The frequency of *SLC20A2* variants in FIBGC (31.3%) was lower than that (50.0%, [5 of 10 families]) in our previous study^8^ because the total number of familial patients has increased. Compared to the data reported by Ramos *et al*.^10^, Ramos *et al*., have screened 177 unrelated probands, and identified SLC20A2 variants at 16.9% (n = 30), followed by PDGFB at 3.4% (n = 6), PDGFRB at 1.7% (n = 3), and XPR1 at 3.4% (n = 6). In Japan, we identified 16 probands (18.6%) carrying either pathogenic or likely pathogenic variants (12.8%, n = 11). *SLC20A2* provided the contribution 8.1% (n = 7), followed by PDGFB at 4.7% (n = 4). We have examined *SLC20A2* in all patients enrolled in the study. We have not identified any pathogenic variants on *PDGFRB* in Japan as far as we examined and have not investigated the genetic variants on *XPR1* and *MYORG*. Two patients with sporadic IBGC who had *PDGFB* variants were identified in our analysis (Case 3, c.33_34delCT; Case 4, c.342_343insC). As *de novo* variants have been reported^26,30^, we should examine the DNA of their parents. A large deletion in the *SLC20A2*^31^, and a partial deletion in the *PDGFB*^27^ were identified. In addition, *XPR1* has been identified as a new causative gene for FIBGC (IBGC6)^6^. Recently biallelic variants in *MYORG* have been reported to cause autosomal recessive IBGC^7^. Genetic studies have been very useful to find key molecules in validating the pathogenesis of the disease. Further genetic studies including other autosomal recessive genes, large deletion or insertion, and copy number variant in IBGC will be needed.

*PDGFB* variants has been reported only in patients with IBGC. As its receptor, PDGFRβ is expressed in the brain microvascular pericytes in the central nervous system. PDGF-B is secreted from the neurons and vascular endothelial cells in the brain^11,32,33^. *PDGFRB* variants cause autosomal-dominant infantile myofibromatosis^34–36^. A point variation in PDGFRB also causes autosomal-dominant Penttinen syndrome^36^. Recently, the overexpression of *PDGFRB* due to the *PDGFRB* variants causes Kosaki overgrowth syndrome^37–40^.

Mice that are deficient in *Pdgfrβ* or *Pdgfb* lack brain pericytes, and impaired maturation of the blood brain barrier is fatal^11^, although heterozygous Pdgfb or Pdgfb knockout mice, as well as double Pdgfb+/−; Pdgfrb+/− mice, did not develop brain calcification^29^. *Pdgfb*-null mice rescued by transgenic re-expression of one human *Pdgfb* allele in only endothelium developed substantial brain calcification at age 1 year^5^. This mouse model is supposed to correspond to the haplo-insufficiency of PDGF-B in patients with *PDGFB* variants. We observed decreased levels in the culture media of the endothelial cells differentiated from the iPS cells established from patients carrying the *PDGFB* variants, as similarly observed in the plasma of the patients. Although the causative role of low plasma PDGF-BB in development of brain calcification still remains unclear, the endothelial cells differentiated from the iPS cells may serve as a cellular model for screening of compounds that are capable of restoring plasma PDGF-BB levels.

Interestingly, PDGF-BB administration stimulates the Pi transport in the aortic vascular smooth muscle cells of rats^19^. Recently, we have reported that Pi levels in patients with IBGC, particularly those with *SLC20A2* variants, are higher than those of the controls^20^. This indicates that the basic mechanism of brain calcification is due to Pi dyshomeostasis in the CSF and interstitial space in the brain. We speculate that the increased Pi levels in the interstitial space and the perivascular space may contribute to the formation of calcification, more precisely mineralization through the formation of complex with Ca and heavy metals^19^. In contrast, the decreased Pi levels in the cell cytoplasm, especially in neurons, is supposed to cause clinical symptoms. On such assumption, 5-ALA has neuroprotective effects against low Pi in neuronal cells^41^. Although some issues should be elucidated, further studies using IBGC-specific iPSCs will give us more information on the pathophysiological mechanism of IBGC in the future. Collectively, appropriate stimulation by PDGF-BB may have beneficial effects on patients with IBGC. The endothelial cells developed from iPSCs of the patients showed a phenotype of the disease and they give us a useful tool for the study on IBGC.

## Materials and Methods

### Participants and samples

We collected clinical information on patients with IBGC in a nationwide study supported by a grant for research on intractable diseases from the Ministry of Health, Labour and Welfare of Japan. The diagnostic criteria were previously described^7^. Briefly, patients with causative biochemical abnormalities, such as high calcium levels, inorganic phosphate, and intact parathyroid hormone, were excluded. The genetic surveys were approved by the Ethics Committee of the Gifu University Graduate School of Medicine and the University of Tokyo. For genetic analysis, 105 patients from 87 hospitals provided written informed consent, and they were then enrolled in the project. Of these patients, 70 came from families with a single affected member, as shown in the available clinical data, and the other 35 patients came from 16 families with multiple members affected. We defined the former as patients with sporadic IBGC and the latter as patients with FIBGC. There were 51 male and 54 female patients. The patients’ mean age ± standard deviation (SD) was 46.7 ± 23.6 years at the time of registration. The 105 patients who were examined included 15 participants from 10 families with *SLC20A2* variants, as revealed in a previous study. All experiments on human blood samples and iPS cells were approved by the Ethics Committees of Gifu University, Gifu Pharmaceutical University, the University of Tokyo and Kyoto University and performed in accordance with Ethical Guidelines for Medical and Health Research Involving Human Subjects in Japan, and Ethical Guidelines for Human Genome/Gene Analysis Research in Japan. After individual written informed consent was obtained, peripheral blood samples were collected. This study was registered to UMIN Clinical Trials Registry approved by International Committee of Medical Journal Editors (UMIN000030100).

### Variant analysis

Genomic DNA was extracted from whole blood samples. In this study, variant analyses was performed by Sanger sequencing of all coding regions, and resequencing strategy was used on the basis of the VariantSEQr platform for the amplification of coding regions and their intron/exon junctions (Applied Biosystems). In the VariantSEQr resequencing system, each PCR primer was designed with priming sites tailed with either the M13 universal forward or reverse sequencing primer for PCR amplification and sequencing reaction under similar PCR and sequencing conditions, as described in detail in the supplementary data (Table e-1). The functional effects of the identified variants were predicted using PolyPhen-2 (http://genetics.bwh.harvard.edu/pph/).

### Transcript analysis

Total RNA was isolated from fresh blood using Tempus^™^ Blood RNA Tube and Tempus^™^ Spin RNA Isolation Kit (Thermo Fisher Scientific, Waltham, MA). For avoiding the amplification of genomic DNA, Absolute RNA Wash Solution (Thermo Fisher Scientific) was used during RNA isolation. cDNA was synthesized using SuperScript^®^III First-standard Synthesis Kit (Thermo Fisher Scientific). To determine the effect of splice site variants, RT-PCR was performed using primers spanning the exon–exon junctions (between exons 2 and 3; exons 4 and 6) and PrimeSTAR^®^ HS DNA Polymerase (Takara Bio Inc.). PCR were separated using a 2% agarose gel. The corresponding bands were purified from the gel using Wizard^®^ SV Gel and PCR Clean-Up System (Promega KK) and were confirmed via Sanger sequencing.

### ELISA

The measurement of human PDGF-BB concentration in the blood was consigned to SRL, Inc. (Tokyo, Japan), using a Human PDGF-BB Quantikine ELISA kit (R&D Systems.), which does not recognize other human PDGFs, including the PDGF-AB heterodimer. iPSC-derived ECs were plated on 96-well plates and cultured for 116 h. Supernatants from iPSC-derived ECs were measured using commercially available ELISA kits (Human PDGF-BB Quantikine ELISA kit [R&D Systems]) in accordance with the manufacturer’s instructions.

### Establishment of iPSCs

Peripheral blood mononuclear cells from patients with IBGC who have PDGF-B variant (c.33_34 delCT, c.160 + 2T > A, and c.457−1G > T) were reprogrammed by inducing the episomal vectors carrying OCT3/4, SOX2, KLF4, L-MYC, LIN28, EBNA1, and p53 carboxy-terminal dominant-negative fragment^42^. The obtained iPSCs were cultured on iMatrix-511 (Nippi, Tokyo, Japan)-coated plates with StemFit medium (Ajinomoto, Tokyo, Japan) supplemented with penicillin/streptomycin. Karyotyping was performed by LSI Medience Corporation (Tokyo, Japan). The genotyping of *PDGF-B* variant was performed by PCR amplification of genomic DNA and direct sequencing (3500xL Genetic Analyzer, Thermo Fisher Scientific). CTK solution was used for harvesting iPSCs, and an embryoid body (EB) was formed. Cell masses were cultured in DMEM/F12 (Thermo Fisher Scientific) comprising 20% knockout serum replacement (KSR, Thermo Fisher Scientific); 2 mM L-glutamine (Thermo Fisher Scientific); 0.1 M of nonessential amino acids (NEAA, Thermo Fisher Scientific); 0.1 M of 2-mercaptoethanol (Thermo Fisher Scientific); and 0.5% penicillin/streptomycin. The medium was replaced every other day, and after 8 days, the EB was cultured for another 8 days in DMEM comprising 10% fetal bovine serum (FBS) on a gelatin-coated coverslip.

### Immunocytochemistry

The cells were fixed with 4% paraformaldehyde and blocked with PBS containing 5% FBS. DAPI (4′,6-diamidino-2-phenylindole) (Thermo Fisher Scientific) was used for labelling the nuclei. Fluorescence imaging was performed using IN CELL Analyzer 6000 (GE Healthcare). The following primary antibodies were used: NANOG (ReproCELL, Yokohama, Japan) (RCAB0003P, 1/500), SSEA-4 (Millipore) (MAB4304, 1/1,000), βIII-tubulin (Millipore) (CBL412, 1/500), SOX-17 (R&D Systems) (AF1924, 1/300), αSMA (DAKO, Glostrup Denmark) (M0851, 1/100), CD31 (Dako) (M023, 1/100), and vWF (Abcam, Cambridge, MA) (Ab6994, 1:400).

### Endothelial cell differentiation

Regarding endothelial differentiation, on day 0, iPSC colonies were dissociated into single cells with Accutase (Sigma). Cells were resuspended in basic medium containing StemPro34 (Invitrogen), L-glutamine (Invitrogen), transferrin (Roche), monothioglycerol (Sigma), and ascorbic acid (Sigma), and Y-27632 (naclai tesque), BMP-4 (Peprotech), and Matrigel (BD) were added to form EBs. On day 1, the medium was supplemented with Activin A (R&D Systems) and BMP4. On day 4, EBs were seeded in Matrigel-coated dishes, and the medium was supplemented with VEGF (R&D Systems) for EC expansion. On day 14, the EBs were harvested using Accutase (Sigma-Aldrich), and CD31 positive cells were positively sorted using magnetic-activated cell sorting using anti-CD31 (Miltenyi) (Fig. 7a).

## Supplementary information

Supplementary Data 1
