# Supplementary material for: Functional evaluation of PDGFB-variants in idiopathic basal ganglia calcification, using patient-derived iPS cells
Source: Sci Rep. 2019 Apr 5;9:5698. doi: 10.1038/s41598-019-42115-y (PMC6450963; doi:10.1038/s41598-019-42115-y)
Supplement: Supplementary file 1 — Supplementary Data 1 [file 41598_2019_42115_MOESM1_ESM.docx]

**Supplementary Information**

Functional evaluation of *PDGFB*-variants in idiopathic basal ganglia calcification, using patient-derived iPS cells

Shin-ichiro Sekine^1,2^, Masayuki Kaneko^1,3^, Masaki Tanaka^4^, Yuhei Ninomiya^1^, Hisaka Kurita^1^, Masatoshi Inden^1^, Megumi Yamada^5^, Yuichi Hayashi^5^, Takashi Inuzuka^5^, Jun Mitsui^4^, Hiroyuki Ishiura^4^, Atsushi Iwata^4^, Hiroto Fujigasaki^6^, Hisamitsu Tamaki^7^, Ryusei Tamaki^8^, Shinsuke Kito^8^, Yoshiharu Taguchi^9^, Kortaro Tanaka^9^, Naoki Atsuta^10^, Gen Sobue^10^, Takayuki Kondo^2,11,12^, Haruhisa Inoue^2,11,12^, Shoji Tsuji^4^, Isao Hozumi^1,5^

^1^ Laboratory of Medical Therapeutics and Molecular Therapeutics, Gifu Pharmaceutical University, Gifu, Japan

^2^ Center for iPS Cell Research and Application, Kyoto University, Kyoto, Japan

^3^ Department of Biochemistry, Institute of Biomedical and Health Sciences, Hiroshima University, Hiroshima, 1-2-3 Kasumi, Minami-ku, Hiroshima, Japan

^4^ Department of Neurology, The University of Tokyo, Tokyo, Japan

^5^ Department of Neurology and Geriatrics, Gifu University Graduate School of Medicine, Japan

^6^ Department of Neurology, Tokyo Metropolitan Bokuto Hospital, Tokyo, Japan

^7^ Department of Pediatrics, Tokyo Metropolitan Bokuto Hospital, Tokyo, Japan

^8^ Department of Neuropsychiatry, Kyorin University Hospital, Tokyo, Japan

^9^ Department of Neurology, Toyama University Hospital, Toyama, Japan

^10^ Department of Neurology, Nagoya University, Nagoya, Japan

^11^ iPSC-based Drug Discovery and Development Team, RIKEN BioResource Research

Center, Kyoto, Japan

^12^ Medical-risk Avoidance based on iPS Cells Team, RIKEN Center for Advanced

Intelligence Project, Kyoto, Japan

Shin-ichiro Sekine, Masayuki Kaneko, and Masaki Tanaka contributed equally to this work. Correspondence and requests for materials should be addressed to I. H. (email: [hozumi@gifu-pu.ac.jp](mailto:hozumi@gifu-pu.ac.jp), Tel & Fax: +81-58-230-8121)

**Mutation analyses**

Mutation analyses were performed by two institutes using method 1 and method 2.

**Method 1:** Genomic DNA was extracted using DNA QuickⅡ(Genomic DNA Separation kit; DS Pharma Biomedical Co., Ltd.). PCR amplification was performed using the primers listed in Supplementary Table 1A (PDGFB) and 1B (PDGFRB). The VariantSEQr-based primers were obtained from the NCBI Probe database. Twenty nanograms of template DNA, 1.2 µl of 10 µM forward and reverse primers (0.6 µM), 3.2 µl of 50% glycerol (8%), and 10 µl of AmpliTaq Gold Fast PCR master mix (Applied Biosystems) were mixed with sterile water up to a total reaction volume of 20 µl. The thermal cycling conditions were as follows: preamplification denaturation (1 cycle), 95 °C for 10 min; amplification (total 40 cycles), 96 °C for 3 s, 68 °C for 13 s; final elongation (1 cycle), 72 °C for 10 s. PCR products were purified with illustra ExoStar (GE Healthcare Life Sciences). The purified PCR products were used for sequencing with an ABI BigDye Terminator v3.1 Cycle Sequencing kit (Applied Biosystems) using M13 forward or reverse primers. After ethanol precipitation, automated sequencing was performed on an ABI 3100 Genetic analyzer using both the forward and reverse primers. Variants found in dbSNP, the 1000 Genomes Project database or the Exome Variant Server database of the National Heart, Lung, and Blood Institute (NHLBI) Exome Sequencing Project were excluded.

**Supplementary Table 1A.** Primer sequences for mutation analysis of *PDGFB*

| Primer | Database name | Sequence (5' to 3') |
| --- | --- | --- |
| Exon1-1･2-F | RSA000980321 | TGTAAAACGACGGCCAGTGATCAGGCGCTCAGGCCTCT |
| Exon1-1-R | RSA000980321 | CAGGAAACAGCTATGACCGGGCGGGAGTTTGCACCTCT |
| Exon1-2-R | RSA001278055 | CAGGAAACAGCTATGACCGGTGCTCGAGCTGCCGTTG |
| Exon1-3-F | RSA001306861 | TGTAAAACGACGGCCAGTGCCTTCCCTTAGAGCCTGTCACC |
| Exon1-3-R | RSA001306861 | CAGGAAACAGCTATGACCGTCGGCATGAATCGCTGCTG |
| Exon2-F | RSA000980318 | TGTAAAACGACGGCCAGTTCGGAGCAGAGCAAAGCGAG |
| Exon2-R | RSA000980318 | CAGGAAACAGCTATGACCCTGCTGGAATCCCAGAGCCC |
| Exon3-F | RSA000994596 | TGTAAAACGACGGCCAGTCAGGTACCAACCCGCCTGCT |
| Exon3-R | RSA000994596 | CAGGAAACAGCTATGACCAGAGGCTTCGGCAGGTCCAG |
| Exon4-F | RSA000980315 | TGTAAAACGACGGCCAGTAGTTCGCTCAGTCCTGAATGTGG |
| Exon4-R | RSA000980315 | CAGGAAACAGCTATGACCTGCTTGGAGGGTCCCTGCTC |
| Exon5-F | RSA000980313 | TGTAAAACGACGGCCAGTGCCTCTCTGGACAGAGCCCA |
| Exon5-R | RSA000980313 | CAGGAAACAGCTATGACCTGGTTCTTGGGTGTAGATCTCATGG |
| Exon6-F | RSA000978682 | TGTAAAACGACGGCCAGTGGGCCTGATCCCATTTCCATT |
| Exon6-R | RSA000978682 | CAGGAAACAGCTATGACCGCGCTCCGGGAATGAGGATA |
| Exon7-F | RSA001278044 | TGTAAAACGACGGCCAGTGTCTCCAAAGCCCACCACCC |
| Exon7-R | RSA001278044 | CAGGAAACAGCTATGACCCATGGCAGGCCTTGGTCAGT |

**Method 2:** Primer sequences for the PCR amplification and sequencing of *PDGFB* and *PDGFRB* gene are listed in Supplementary Table 2A and B. Twenty ng of template DNA, 1.2 µl of forward and reverse primers (3 µM), 0.2 µl of LA Taq polymerase (TaKaRa), 2 µl of PCR buffer, 2 µl of MgCl_2_ (25 mM), and 3.2µl of dNTP mixture (2.5 mM each) were mixed with dH_2_O up to a total reaction volume of 20 µl. Thermal cycling conditions were as follows: preamplification denaturation (1 cycle), 98 °C for 2 min; amplification (total 35 cycles), 98 °C for 10 s, 62 °C (first 5 cycles), 60 °C (next 5 cycles) or 58 °C (last 25 cycles) for 30 s (2.5 min for PDGFRB exon 12), 68 °C for 30s; final elongation (1 cycle), 68 °C for 5min. PCR products were purified with ExoSAP-IT^TM^ (USB Corporation). The cleaned PCR products were used for sequencing with the ABI BigDye^®^ Terminator v3.1 Cycle Sequencing Kit (Applied Biosystems) followed by clean-up of products with BigDye XTerminator^®^ Purification Kit (Applied Biosystems). Automated sequencing was performed using an ABI 3730 DNA analyzer (Applied Byosystems).

**Supplementary Table 2A.** Primer sequences for mutation analysis of *PDGFB*.

| Primer | Sequence (5' to 3') |
| --- | --- |
| PDGFB_1F | GAGTGAAGACGAACCATCGAC |
| PDGFB_1R | CGCTGTTGCCTTCCCTTAGA |
| PDGFB_2F | CCTGTGACCTTGGAGCTTTC |
| PDGFB_2R | CAAGTCCCAGGTACCAACCC |
| PDGFB_3F | GGGACTGTTCTCTGGAGTGG |
| PDGFB_3R | GAGTTGTAAGAGGACCCTCGG |
| PDGFB_4F | TTGAAGGGCGTGAGAAAGAG |
| PDGFB_4R | AAGCCTGGTCAGGTATGAGC |
| PDGFB_5F | AGGTCCTGCTATTGTTTGGG |
| PDGFB_5R | AGGGAGGAACCTGGCTTG |
| PDGFB_6F | gagagacctccccaatggtc |
| PDGFB_6R | ctttcccctgaccccatc |

Supplementary Fig. 1. DNA sequence electropherograms showing different heterozygous mutations in *PDGFB* identified in IBGC patients. Letters in red indicate mutation sequences.


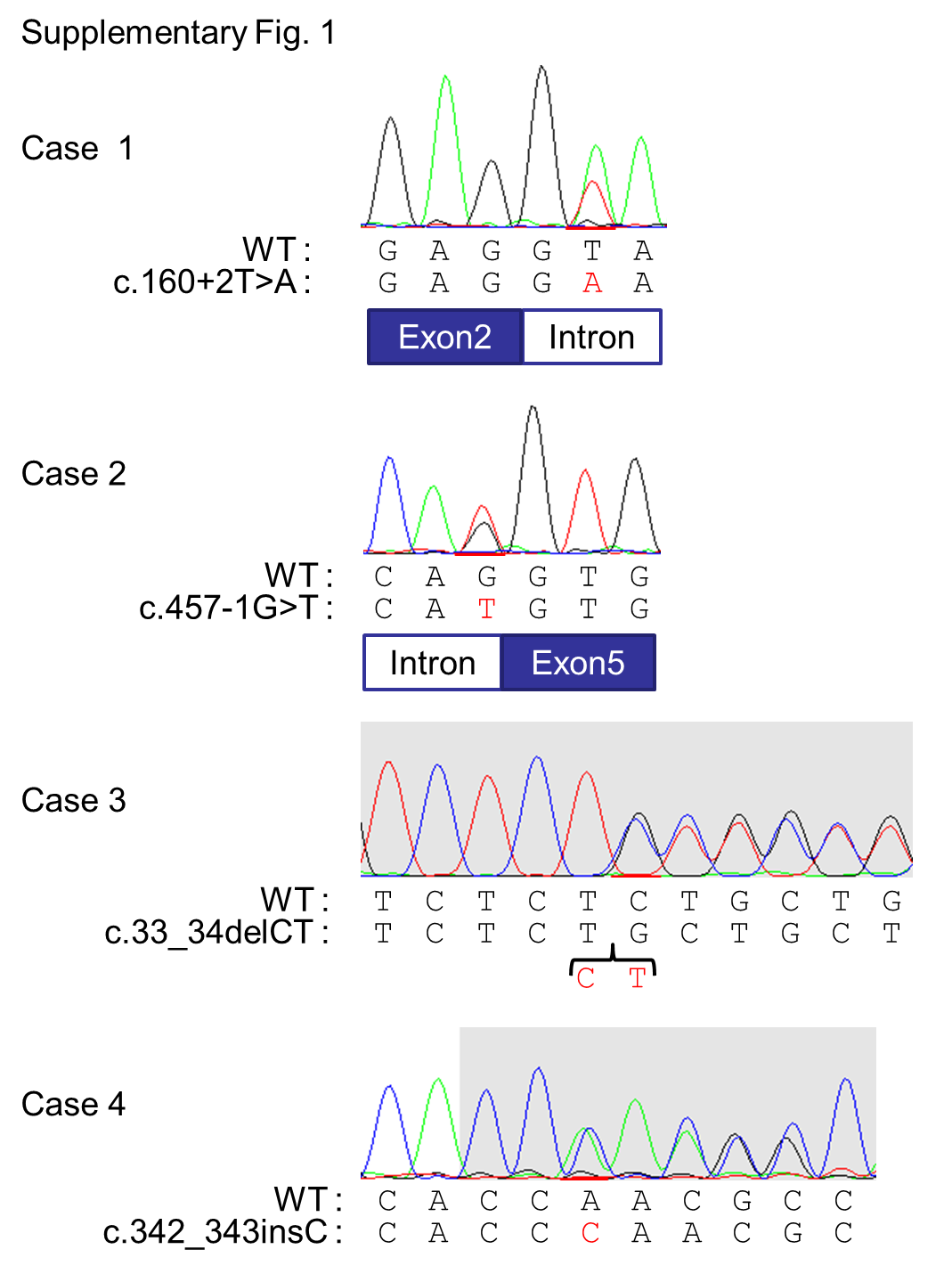


**Supplementary Fig. 2**


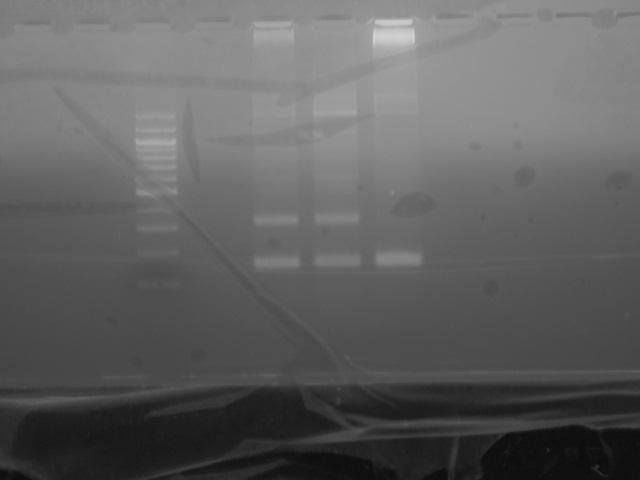
**A**

1. Full-length gel of Fig. 5A is presented above. The portion surrounded by a blue square is presented in Fig. 5A.


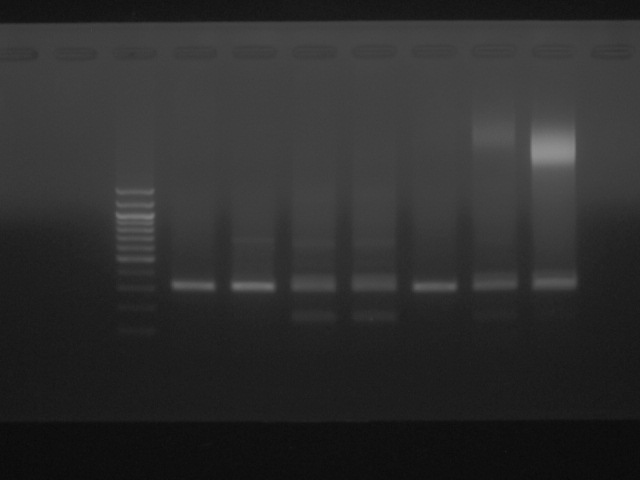
**B**

(B) Full-length gel of Fig. 5C is presented above. The portion surrounded by a blue square is presented in Fig. 5C.
